# Screen Time, Child Depression, and Anxiety During the COVID-19 Pandemic: Systematic Review and Meta-Analysis

**DOI:** 10.2196/83228

**Published:** 2026-04-01

**Authors:** Marissa Yoshizawa, Jennifer Rafeedie, Jasmyn J Tang, Bryan T Lei, Ramon Durazo-Arvizu, Danny Azucar, Sharon Hudson, Sheela Rao, Karen Kay Imagawa, Alexis Deavenport-Saman

**Affiliations:** 1 Department of Pediatrics, Children's Hospital Los Angeles, 4650 Sunset Blvd MS53, Los Angeles, CA, 90027, United States, 1 323 361 5734, 1 323 361 1383; 2 Department of Pediatrics, Keck School of Medicine, University of Southern California, Los Angeles, CA, United States; 3 Division of General Internal Medicine and Health Services, David Geffen School of Medicine, University of California Los Angeles, Los Angeles, CA, United States; 4 Department of Pediatrics, The Saban Research Institute, Children's Hospital Los Angeles, Los Angeles, CA, United States; 5 AltaMed Institute for Health Equity, Los Angeles, CA, United States

**Keywords:** screen time, anxiety, depression, COVID-19 pandemic, children, adolescents, mental health

## Abstract

**Background:**

In response to the COVID-19 pandemic, governments around the world enforced stay-at-home orders and social distancing guidelines that amplified the use of screen time among pediatric populations. Excessive screen time may negatively impact mental health by increasing depression and anxiety.

**Objective:**

The first aim was to conduct a systematic review of articles examining screen time and mental health outcomes among children and adolescents during the COVID-19 pandemic from 2020 to 2023. The second aim was to determine the combined effect sizes for the associations of screen time and depression and/or anxiety among children and adolescents during the COVID-19 pandemic from 2020 to 2023 and whether gender or age influenced outcomes.

**Methods:**

Bibliographic databases were searched including MEDLINE (Ovid), Embase (Elsevier), Cochrane Library (Wiley), CINAHL Complete (EBSCO), and PsycINFO (EBSCO). There were a total of 6462 nonduplicate studies that were screened. Study inclusion criteria included children ages 0 to <18 years, the effects of screen time on children during the COVID-19 pandemic, screen time and depression and/or anxiety, articles written in English, and articles, including quantitative and qualitative studies, published between 2020 and 2023. A total of 452 articles underwent full-text review with 23 articles meeting criteria for final article extraction.

**Results:**

A total of 23 studies totaling 29,581 children and adolescents were included in the study. Results showed that most studies reported a positive association between screen time and depression and/or anxiety (*r*=0.175, 95% CI 0.124-0.226, *P*<.001 and *r*=0.157, 95% CI 0.0994-0.214, *P*<.001, respectively) during COVID-19. Meta-regression revealed that screen time measured in problematic use of electronic devices had a 0.15 higher correlation with anxiety compared to screen time measured in duration of electronic device use.

**Conclusions:**

During the COVID-19 pandemic, children and adolescents with higher levels of screen time had increased depression and/or anxiety. Findings suggest the need for ongoing parent, professional, and self-monitoring of youth screen behaviors and habits as well as activities that promote social connectedness during global or national health emergencies.

## Introduction

The global COVID-19 pandemic impacted children’s screen time. As numerous restrictions were put on children and adolescents during COVID-19, regular screen usage increased due to the closure of regular outdoor activities and increase in online learning. A meta-analysis of over 29,000 children showed a 52% increase in screen time, particularly for those aged 12‐18 years [1]. Excessive screen time increased, with adolescents reported to have almost 6 hours per day of screen use, mainly spent on watching shows, movies, and playing games [2]. One-third of adolescents aged 13‐17 years reported using social media “almost constantly” [3]. Children at 9.5 years of age who were surveyed during the COVID-19 pandemic reported an average increase in recreational screen time of 11 hours per week compared to the 1 hour per week increase reported in prepandemic surveys conducted at ages 5 and 8 years [4].

The COVID-19 pandemic also exacerbated the preexisting mental health challenges of children and adolescents [5]. A meta-analysis covering over 80,000 youths globally found that the prevalence of anxiety and depression doubled during the COVID-19 pandemic to 20%‐25% [6]. In the United States, emergency visits for children due to mental health problems rose drastically by 31%‐50% since the beginning of the pandemic [7]. Globally, depression and posttraumatic stress disorder were significantly more prevalent among children aged 7 to 15 years [8] due to the separation from classmates and friends. Overall, distancing measures during the pandemic were harmful to adolescent mental health [9].

The opportunity for excessive screen use during the COVID-19 pandemic also impacted mental health outcomes among children and adolescents. Social media and media addiction were associated with ill-being among adolescents [9]. The US Surgeon General released a report [10] indicating potential risks of social media use on the mental health of children and adolescents and called for more evidence. During the pandemic, a greater duration of screen time (such as television viewing [10] or digital media time [10], including social media use [11,12]) was associated with higher levels of depression and anxiety [12]. The associations between screen time and poor mental health outcomes have been found in individual studies; however, to our knowledge, there have not been meta-analyses of screen time using standardized or validated measures of depression and anxiety during the pandemic.

Thus, this review aims to fill this gap by examining the mental health effects of screen time in children and adolescents throughout the COVID-19 pandemic. First, the purpose was to conduct a systematic review of articles examining screen time and depression and anxiety among children and adolescents during the COVID-19 pandemic from 2020 to 2023. Second, the purpose was to determine the combined effect size for the association of screen time and depression and anxiety among children and adolescents during the COVID-19 pandemic from 2020 to 2023. We additionally explored the influence of gender and age on the relationship between screen time and mental health outcomes.

## Methods

### Eligibility Criteria

Studies identified through database searches were screened for the following inclusion criteria: (1) children ages 0 to <18 years, (2) examination of the effects of screen time on children during the COVID-19 pandemic (eg, video games, smartphone use, or computer use), (3) examination of screen time and anxiety and/or depression, (4) articles were written in English, and (5) studies, including quantitative and qualitative studies, published between 2020 and 2023. Studies were excluded if they did not describe the effects of screen time on children during the COVID-19 pandemic (eg, provider-led and provider-care interventions; telehealth) or did not meet the inclusion criteria listed above.

### Information Sources and Search Strategy

We initially searched the following bibliographic databases: MEDLINE (Ovid), Embase (Elsevier), Cochrane Library (Wiley), CINAHL Complete (EBSCO), and PsycINFO (EBSCO). A MEDLINE search strategy was created using a combination of Medical Subject Headings (MeSH) and keywords for the concepts of COVID-19, screen time, and pediatrics. All team members reviewed the strategy and results; with team approval, the search was customized using controlled vocabulary (when available) and keywords in Embase, Cochrane Library, and PsycINFO (Multimedia Appendix 1). As of November 2021, all resulting citations were exported into an EndNote 20 (Clarivate Analytics) library with duplicates removed [13]. The resulting collection of citations was then imported into Covidence for screening. In August 2022, the search was run again in all bibliographic databases and any unique citations were imported into Covidence. Finally, in February 2023, the search was rerun given the refinement of the research question to include validated measures of depression and anxiety and to capture new studies that had been recently published. This was done again in MEDLINE and PsycINFO and also included CINAHL. All articles were imported and screened in Covidence.

### Data Collection Process

Two reviewers independently extracted data from each article and the extracted data were sent to the research team for consensus. The following data were extracted from each study: the first author, the date and place of publication, the number of participants enrolled, participant demographics, measures/assessments used, study start and end date, inclusion criteria, findings summary, and effect sizes. The extracted data were collected in Covidence. Any disagreements were resolved in discussions with the research team.

Screen time during the COVID-19 pandemic, including problematic use that was excessive or disordered (eg, video games, smartphone or computer use, social media use), was defined through self-reports of screen time duration. Anxiety was defined using self-report standardized questionnaires as well as standardized parent report measures on anxiety symptoms. Depression was defined using self-report standardized questionnaires as well as standardized parent report measures on depressive symptoms.

### Study Risk of Bias Assessment

The STROBE (Strengthening the Reporting of Observational studies in Epidemiology) checklist was used to assess the quality of included studies on a scale from 0 to 21 (Table 1) [14]. Two reviewers assessed each study independently and then met to achieve consensus.

**Table 1.** STROBE (Strengthening the Reporting of Observational studies in Epidemiology) checklist for bias assessment.

	Introduction		Methods				Results		Discussion		
ID	Background	Objectives	Participants	Variables	Data/measures	Statistical methods	Descriptive data	Outcomes	Results	Limitations	Total
1 [15]	2	2	2	2	2	2	2	2	2	2	20
2 [16]	2	2	2	2	2	2	2	2	2	2	20
3 [17]	2	2	2	2	2	2	2	2	2	2	20
4 [18]	2	2	2	2	2	2	2	2	2	2	20
5 [19]	2	2	2	2	2	0	2	2	2	2	18
6 [20]	2	2	2	2	2	2	2	2	2	2	20
7 [21]	2	2	2	2	2	1	2	2	2	2	19
8 [22]	2	2	2	2	2	2	2	2	2	2	20
9 [23]	2	2	2	2	2	2	2	2	2	2	20
10 [24]	2	2	2	2	2	2	2	2	2	2	20
11 [25]	2	2	2	2	2	1	2	2	2	2	19
12 [26]	2	2	2	2	2	2	2	2	2	2	20
13 [27]	2	2	2	2	2	2	2	2	2	2	20
14 [28]	2	2	2	2	2	2	2	2	2	2	20
15 [29]	2	2	2	2	2	2	2	2	2	2	20
16 [30]	2	2	2	2	2	2	2	2	2	1	19
17 [30]	2	2	2	2	2	2	2	2	2	2	20
18 [11]	2	2	2	2	2	2	2	2	2	2	19
19 [31]	2	2	2	2	2	2	2	2	2	2	20
20 [32]	2	2	2	2	2	2	2	2	2	2	20
21 [33]	2	2	2	2	2	2	2	2	2	2	20
22 [34]	2	2	2	2	2	1	2	2	2	2	19
23 [35]	2	2	2	2	2	1	2	2	2	2	19

### Effect Measures, Synthesis Methods, and Analyses

Fisher *r*-to-*z* transformation was used as the effect measure to quantify the relationship between screen time, anxiety, and depression, and results were converted back to Pearson correlations for interpretation. Studies were excluded if they were missing sufficient summary statistics and/or were unconvertible into appropriate effect sizes. Studies reported in other effect measures were converted to correlations using the conversion formulas proposed by Borenstein et al [14]. If a study assessed the same outcome with more than one measure, the average effect size was used in the analysis [36].

A random-effects model was used for all meta-analysis to account for heterogeneity across studies. Pooled correlations of *r*=0.10, *r*=0.30, and *r*=0.50 were considered as small, medium, and large effect sizes, respectively [37]. Heterogeneity (ie, $\tau^{2}$) was assessed using the restricted maximum-likelihood estimator. In addition to the estimation of $\tau^{2}$, the *Q* test for heterogeneity and the *I*^2^ statistics are also reported [38]. Forest plots were generated for each outcome to visualize individual study effects and pooled estimates. Funnel plots were used to detect potential publication bias. Meta-regression analysis was performed to assess the impact of age, gender, and the definition of screen time (duration of use vs problematic use) on the effect sizes [39]. To evaluate the robustness of the results, sensitivity analysis was conducted by excluding studies identified as potential outliers or exerting excessive influence on the pooled effect using studentized residuals and the Cook distance [40]. All statistical analyses were performed using RStudio (version 4.4.1; Posit PBC) and the *metafor* package [41].

## Results

### Search Results and Included Studies

**Figure 1.**
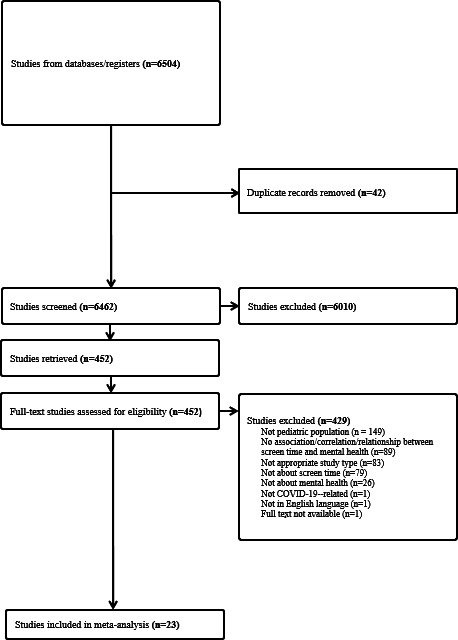
PRISMA flowchart for studies of screen time during the COVID-19 pandemic and pediatric mental health. PRISMA: Preferred Reporting Items for Systematic Reviews and Meta-Analyses.

In the initial database search, 6504 records were obtained. After duplicate removal, the titles and abstracts of 6462 records were screened independently by 7 coders based on the predefined eligibility criteria. After the title and abstract screening, 452 articles were retained. Following full-text screening, 429 articles were excluded from the systematic review, and 23 articles totaling 29,581 children and adolescents were retained in the systematic review and meta-analysis. See Figure 1 for the PRISMA (Preferred Reporting Items for Systematic Reviews and Meta-Analyses) flow diagram of observational studies.

### Study Characteristics

Across the 23 studies (Table 2), children and adolescents were 8 to <18 years of age, with a mean age of 13.54 (SD 0.43) years; in addition, 49% (n=14,390) were male and 51% (n=15,191) were female. Of the 23 studies, 39% (n=9) were from North America, 44% (n=10) were from Asia, and 17% (n=4) were from Europe. Studies were published globally in the following countries: United States (n=6), China (n=6), Canada (n=3), Hong Kong (n=1), Hungary (n=1), Italy (n=2), India (n=1), Iran (n=1), Poland (n=1), and South Korea (n=1). Of the 23 studies, 96% (n=22) examined depression, 83% (n=19) examined anxiety, and 78% (n=18) examined both depression and anxiety.

**Table 2.** Characteristics of studies.

ID	Author	Country	Sample size (N)	Male (%)	Age (years)	Screen time	Depression	Anxiety
1a	Alves et al, 2021 [15]	United States	30	43	11.7	Duration	PANAS-C^a^	STAIC^b^
1b	Alves et al, 2021 [15]	United States	34	32	11.9	Duration	PANAS-C	STAIC
2	Berki et al, 2021 [16]	Hungary	705	37	15.9	Duration	CDI^c^	N/A
3	Charmaraman et al, 2022 [17]	United States	900	47	12.53	Duration	CESDR-10^d^	SADS^e^
4	Chen et al, 2021 [18]	China	1357	50	10.71	Duration	DASS-21^f^	DASS-21
5	De Pasquale et al, 2021 [42]	Italy	162	48.2	9.4	Problematic use	N/A	CAM-S^g^
6	Ellis et al, 2020 [19]	Canada	1054	21.9	16.68	Duration	BSI^h^	N/A
7	Fazeli et al, 2020 [20]	Iran	1512	56.4	15.51	Problematic use	DASS	DASS
8	Lee et al, 2021 [21]	South Korea	264	100	17.57	Problematic use	CES-D^i^	N/A
9	Liu et al, 2022 [22]	China	5581	48.5	13.8	Problematic use	SDS^j^	SAS^k^
10	McArthur et al, 2021 [23]	Canada	846	52.8	9.85	Duration	BASC-3^l^	BASC-3
11	Moitra et al, 2021 [24]	India	1298	53.3	13.2	Duration	PHQ^m^-2	N/A
12	Mousavi et al, 2022 [25]	United States	215	51.2	15.63	Duration	DASS	DASS
13	Murata et al, 2021 [26]	United States	583	20	15.8	Duration	PHQ-9	GAD^n^ -7
14	Muzi et al, 2021 [27]	Italy	62	37	15.43	Problematic use	YSR^o^ 11‐18	YSR 11‐18
15	Ren et al, 2021 [28]	China	1771	51.8	N/A	Duration	PHQ-9	GAD-7
16	Sikorska et al, 2021 [29]	Poland	370	32.7	15.38	Duration	DASS-21	DASS-21
17a	Tandon et al, old, 2021 [30]	United States	500	50.5	14	Duration	SDQ^p^	SDQ
17b	Tandon et al, young, 2021 [30]	United States	500	52.6	8	Duration	SDQ	SDQ
18	Tao et al, 2021 [11]	United States	407	17.7	16.47	Duration	CES-D	GAD-7
19	Tardif-Grenier et al, 2021 [31]	Canada	895	26.3	14.69	Duration	CES-D	SCARED-R^q^
20	Teng et al, 2021 [32]	China	1778	50.7	N/A	Duration	CES-D	STAI^r^
21	Xiang et al, 2022 [33]	China	2423	51.2	10.5	Duration	DASS-21	DASS-21
22	Zhang et al, 2022 [34]	China	3471	51.6	14.1	Duration	PHQ-9	GAD-7
23	Zhu et al, 2021 [35]	Hong Kong	2863	59	12.6	Problematic use	PHQ-9	GAD-7

a PANAS-C: Positive and Negative Affect Scale - Child Form.

b STAIC: State-Trait Anxiety Inventory for Children.

c CDI: Children’s Depression Inventory.

d CESDR-10: Center for Epidemiologic Studies Depression Scale Revised.

e SADS: Social Avoidance and Distress Scale.

f DASS-21: Depression Anxiety Stress Scale.

g CAM-S: Child Anxiety Meter-State.

h BSI: Brief Symptom Inventory.

i CES-D: The Center for Epidemiologic Studies Depression Scale.

j SDS: Self-Rating Depression Scale.

k SAS: Self-Rating Anxiety Scale.

l BASC-3: Behavior Assessment System for Children-Third Edition.

m PHQ: Patient Health Questionnaire.

n GAD-7: Generalized Anxiety Disorder 7-item.

o YSR: Youth Self-Report.

p SDQ: Strengths and Difficulties Questionnaire.

q SCARED-R: Screen for Child Anxiety Related Disorders-Revised.

r STAI: State Trait Anxiety Inventory.

### Risk of Bias in Studies

The STROBE checklist [14] was used to assess the quality of studies and any potential bias as seen in Table 1. Two independent reviewers assessed each study, resulting in a possible score from 0 to 20. Each section of the STROBE checklist had a potential score of 0 to 2, with 0 meaning criteria were not met, 1 meaning criteria were partially met, and 2 meaning criteria were fully met. Two reviewers ranked the scores, and any disagreements were discussed with a third reviewer to achieve consensus. All studies used validated questionnaires for depression and anxiety. McArthur et al [23] used a large longitudinal cohort, which allowed for examination of differences over time. The majority of studies were cross-sectional and were conducted during the global pandemic, which did not allow for examination of causal relationships. The following studies were found to have a lower quality assessment. De Pasquale et al [42] did not provide any information on the statistical tests that were conducted in the methods. Fazeli et al [20] had limited information on the statistical methods. Moitra and Madan [24] conducted a mediation analysis on a cross-sectional study but did not indicate the temporal order in their survey methods. Zhu et al [35] imputed more than 25% of missing data. Sikorska et al [29] did not adequately address potential limitations.

### Screen Time and Depression

**Figure 2.**
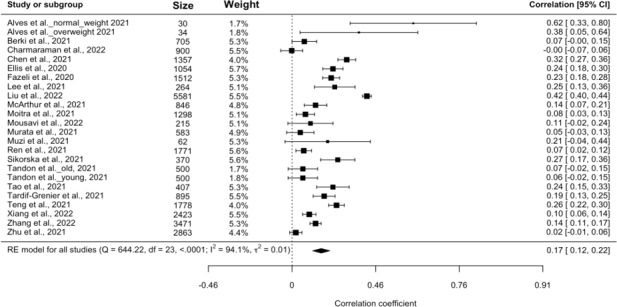
Forest plot for depression.

Twenty-two of the 23 included studies (all except [42]) reported comparable effect sizes for screen time and depression. Two studies [15,30] presented results only for stratified subgroups rather than an overall sample. Therefore, we treated the stratified groups as separate studies in the analysis and increased the sample size to 24 for the analysis. The observed correlation coefficients ranged from −0.00160 to 0.620. The random-effects model revealed a significant, positive, small estimated pooled correlation (*r*=0.175, 95% CI 0.124-0.226; *P*<.001; *I*^2^=94%). According to the *Q* test, the true estimates appear to be heterogeneous (*Q*_23_=644.22, *P*<.001, $\tau^{2}$=0.0138, *I*^2^=95%). A forest plot showing the observed and the estimated correlation is shown in Figure 2 [11,15-31,33-35]. Meta-regression showed no significant associations of depression with age and gender.

**Figure 3.**
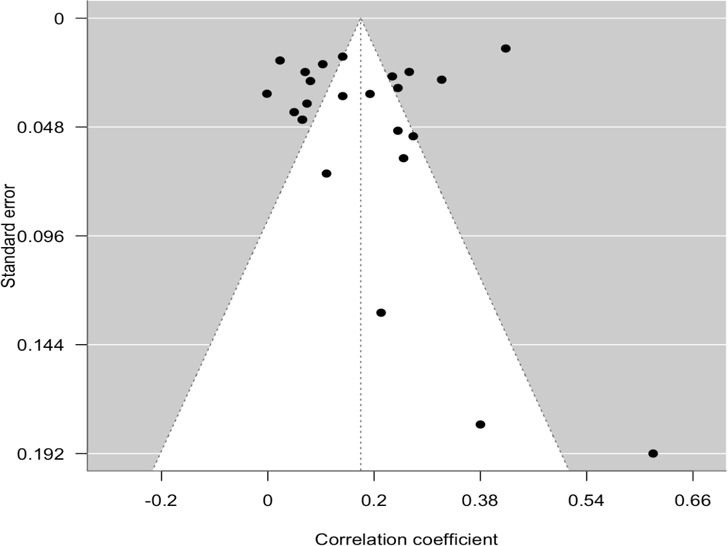
Funnel plot for depression.

A funnel plot of the estimates is shown in Figure 3. The plot is reasonably symmetrical around the pooled estimate, indicating no strong evidence of publication bias or small-study effects. Both the Cook distance and studentized residuals showed that one study [22] could be overly influential. Sensitivity analysis was conducted by removing the study. The result from the sensitivity analysis remained stable after removing the potential outlier (*r*=0.158, 95% CI 0.114-0.202; *P*<.001), supporting the result from the main analysis. Although *I*^2^ decreased from 95% to 90%, heterogeneity remained high across studies.

### Screen Time and Anxiety

**Figure 4.**
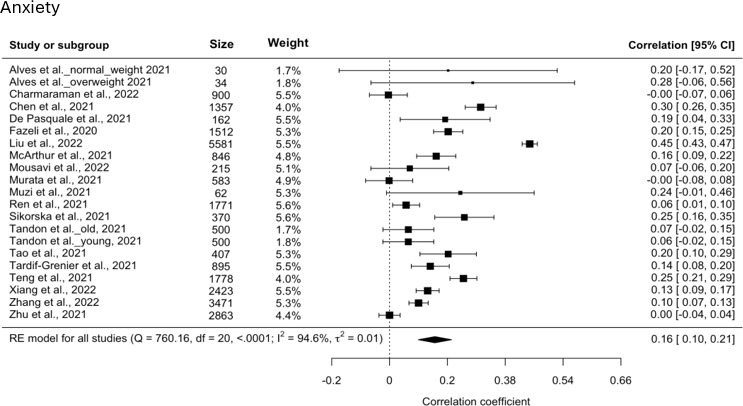
Forest plot for anxiety.

A total of 19 studies reported correlation coefficients for screen time and anxiety, ranging from −0.00390 to 0.450. Since the results from Alves et al [15] and Tandon et al [30] were reported as two stratified subgroups, it increased the total sample size to 21 for the analysis. A random-effects model for screen time and anxiety again indicated a significant yet small estimated pooled correlation (*r*=0.157, 95% CI 0.0994-0.214; *P*<.001), with a *Q* test (*Q*_20_=760.16, *P*<.001,$\tau^{2}$=0.0150, *I*^2^=95%) demonstrating high heterogeneity. A forest plot showing the observed and estimated correlations is shown in Figure 4 [11,15,17,18,20,22,23,25-30,33-35,42]. Meta-regression revealed that compared to studies where screen time was measured as duration of use, those defining it as problematic use have an average 0.1585 higher Fisher *z* score for the correlation between screen time and anxiety. In terms of correlation, this translates to roughly a 0.15 increase, indicating a stronger correlation with anxiety in studies with problematic use.

### Certainty of Evidence

**Figure 5.**
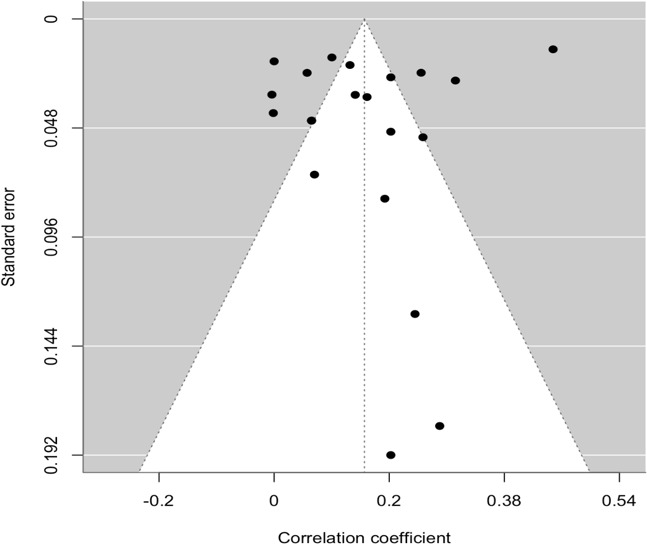
Funnel plot for anxiety.

The funnel plot in Figure 5 was fairly symmetrical around the pooled effect size visually, with no indication of publication bias or small-study effects. The same study [22] was considered to be an outlier based on the Cook distance and studentized residual. The result from the sensitivity analysis yielded a slightly lower but still significant correlation (*r*=0.135, 95% CI 0.0890-0.181; *P*<.001), supporting the result from the main analysis. Although *I*^2^ decreased from 95% to 89%, heterogeneity remained high overall, suggesting the true effect varies across different studies.

## Discussion

### Principal Findings

This meta-analysis of a total of 23 global studies examined screen time and mental health outcomes among 29,581 children and adolescents during the COVID-19 pandemic. Screen time had a significant, positive, small estimated pooled correlation with increased depression (22 studies) and increased anxiety (19 studies). Problematic screen use that was excessive or disordered had a stronger correlation with anxiety than average screen use. Age and gender did not influence mental health outcomes; most youth were adolescents and gender was equally distributed. Sensitivity analyses for studies with outliers for depression and anxiety supported the results from the main analysis.

Although various studies examined different types of screen time during the pandemic, results remained the same. Studies reported greater time spent on social media was associated with higher depressive [11,19] and anxiety symptoms [11]. Zhu et al [35] reported pathological gaming behavior (as compared to excessive, leisure, and none) was associated with increased depression and anxiety. Berki et al [16] reported that more hours of daily screen time (time spent using electronic devices such as a computer, tablet, or smartphone) were associated with higher depressive symptoms. Studies also found that a greater number of hours spent on video games and social media predicted higher depressive [18,26,43] and anxiety [18] symptoms and negative perceived well-being [44].

### Strengths

There were various strengths in this meta-analysis. A rigorous process was implemented, with multiple reviewers using a systematic approach to achieve consensus when screening studies for eligibility and extracting data from studies that met eligibility criteria. Additionally, depression and anxiety were measured using validated questionnaires, and the meta-analysis methodology provided precision in the estimate of the effect size, which increased the validity and generalizability of the findings. Extracted studies included pooled data from 10 countries in North America, Europe, and Asia, further increasing the generalizability of the findings. Sensitivity analyses demonstrated the robustness of the study results. Finally, the COVID-19 pandemic was a global emergency that served as a natural experiment and magnified the conditions to allow youth to participate in excessive screen time, providing a rare opportunity with high statistical power to examine the impact on mental health outcomes.

### Limitations

There were limitations in this study. Most studies examined the negative impact of screen time, which may have introduced some bias. Given the unprecedented nature of the global pandemic, most studies were cross-sectional, allowing for the examination of the relationship between screen time and increased depression or anxiety at one point in time. The results are not generalizable to all age groups as the cohort mostly consisted of adolescents, with a mean age of 13.54 years. More information is needed about how the COVID-19 pandemic impacted school-age (6‐12 year olds) and young children (0‐5 year olds). Screen time was also measured through youth self-reports or parent reports, which may not accurately reflect the amount of time spent on screens due to recall bias. It is possible that other factors aside from screen time, such as the adjustment to the online schooling format and/or cybervictimization, may have impacted anxiety and depression. Since this study included data from multiple countries, there may have been different methods for collecting and tracking data, and it is possible that some mental health data could have been underreported.

### Clinical Implications

During the pandemic, children and adolescents with increased screen time were negatively impacted in terms of depression and anxiety due to higher levels of screen time. These findings suggest the need for ongoing assessment of children and adolescents’ quality and quantity of screen behaviors to identify risk factors to prevent or treat mental health outcomes, especially in the event of another global or national emergency. More research is needed to develop a validated tool to assess screen time [45,46]. Given the significance of screen time in the lives of youth, it is important to consider how to address unhealthy media habits that may increase during a pandemic. The American Academy of Pediatrics recommends the 5 Cs of Media Use to promote healthy media habits [47]. This includes Child, Content, Calm, Crowding Out, and Communication. Practitioners should work with families to develop family media plans that address rules around screen time, balancing family and media time, conversations around responsible social media use, teaching emotion regulation strategies, parental modeling of screen use, and monitoring content for quality, developmental appropriateness, and privacy. Additionally, pediatricians can provide anticipatory guidance to help children and families prepare for emergencies, such as a global pandemic [48]. Children and adolescents should learn from an early age about the benefits and risks of screen use, with an emphasis on developing healthy screen habits that maximize positive social interactions, learning, and recreation; increasing awareness of safety factors such as the protection of privacy; and creating self-awareness that will guard against the negative impacts on mental health related to screen overuse. Finally, promoting positive childhood experiences during a global emergency may help to buffer negative impacts of excessive screen time on mental health outcomes by nurturing healthy social-emotional development.

### Conclusion

This meta-analysis includes the evidence from 23 studies on screen time and mental health. During the COVID-19 pandemic, children and adolescents with higher levels of screen time had increased depression and/or anxiety. These data suggest the need for ongoing parent, professional, and self-monitoring of youth screen behaviors and habits as well as activities that promote social connectedness during global or national health emergencies.

## Supplementary material

10.2196/83228 Multimedia Appendix 1 Search strategies by database.

10.2196/83228 Checklist 1 PRISMA checklist.

## Abbreviations

PRISMA: Preferred Reporting Items for Systematic Reviews and Meta-Analyses
STROBE: Strengthening the Reporting of Observational studies in Epidemiology
