# Supplementary material for: Screen Time, Child Depression, and Anxiety During the COVID-19 Pandemic: Systematic Review and Meta-Analysis
Source: JMIR Pediatr Parent. 2026 Apr 1;9:e83228. doi: 10.2196/83228 (PMC13041624; doi:10.2196/83228)
Supplement: Multimedia Appendix 1 [file pediatrics-v9-e83228-s001.docx]

Search Strategies by Database

| **Database Name:**  Medline  **Database Vendor:**  Ovid  **Database Coverage:**  1946 – Present  **Date Last Searched:**  February 16, 2023  **Limits:**  yr="2020-Current"  **Results:** 1,952 | (exp COVID-19/ OR exp SARS-CoV-2/ OR Pandemics/ OR ("COVID 19" OR COVID19 OR "2020 nCoV" OR Coronavirus Disease 19 OR 2020 Novel Coronavirus OR "SARS CoV 2" OR SARS Coronavirus 2 OR Wuhan Coronavirus OR Wuhan Virus OR Severe Acute Respiratory Syndrome Coronavirus 2 OR pandemic OR pandemics).af)  AND  (Screen Time/ OR Video Games/ OR Internet Addiction Disorder/ OR exp Cell Phone/ OR "Cell Phone Use"/ OR Social Media/ OR Online Social Networking/ OR Blogging/ OR Mobile Applications/ OR exp Computer Systems/ OR exp User-Computer Interface/ OR Attitude to Computers/ OR exp Videoconferencing/ OR exp Television/ OR Motion Pictures/ OR Multimedia/ OR Electronic Mail/ OR Education, Distance/ OR screentime.mp OR "screen time".mp OR "screen times".mp OR "video game".mp OR "video games".mp OR "computer game".mp OR "computer games".mp OR xbox.mp OR "x box".mp OR playstation.mp OR "play station".mp OR nintendo.mp OR pokemon.mp OR fortnite.mp OR (mobile ADJ7 app).mp OR (mobile ADJ7 apps).mp OR "cell phone".mp OR "cell phones".mp OR cellphone.mp OR cellphones.mp OR "smart phone".mp OR "smart phones".mp OR smartphone.mp OR smartphones.mp OR iphone.mp OR iphones.mp OR "text messag*".mp OR textmessag*.mp OR whatsapp.mp OR texting.mp OR "social media".mp OR "social network*".mp OR facebook.mp OR "face book".mp OR instagram.mp OR tiktok.mp OR "tik tok".mp OR twitter.mp OR snapchat.mp OR newsfeed.mp OR "news feed".mp OR ipad.mp OR ipads.mp OR zoom.mp OR webex.mp OR (microsoft teams).mp OR webconferenc*.mp OR "web conferenc*".mp OR videoconferenc*.mp OR "video conferenc*".mp OR facetim*.mp OR "google hangout".mp OR "google hangouts".mp OR television.mp OR televisions.mp OR tv.mp OR tvs.mp OR livestream*.mp OR "live stream*".mp OR laptop.mp OR laptops.mp OR macbook.mp OR email.mp OR emails.mp OR "e mail".mp OR "e mails".mp OR (distance ADJ7 (learn* OR educat* OR school* OR instruct*)).mp OR (remote ADJ7 (learn* OR educat* OR school* OR instruct*)).mp OR (virtual OR online).tw)  AND  (exp Child/ OR Adolescent/ OR Minors/ OR exp Parents/ OR exp Pediatrics/ OR School Teachers/ OR *Schools/ OR Early Intervention, Educational/ OR *Child Care/ OR *Child Rearing/ OR toddler.af OR toddlers.af OR "pre school*".af OR preschool*.af OR pre-k.af OR child*.af OR kid.af OR kids.af OR juvenile.af OR juveniles.af OR minors.af OR youth.af OR youths.af OR youngster.af OR youngsters.af OR daycare.tw OR daycares.tw OR "day care".tw OR "day cares".tw OR kindergarten*.af OR "elementary school*".af OR "grade school*".af OR preadolescen*.af OR "pre adolescen*".af OR preteen*.af OR "middle school*".af OR adolescen*.af OR teen*.af OR "high school*".af OR "secondary school*".af OR parent.af OR parents.af OR parental.af OR mother.af OR mothers.af OR father.af OR fathers.af OR pediatric*.af OR paediatric*.af OR (early ADJ5 educat*).af OR (early ADJ5 intervent*).af)  AND  Depression/ OR Anxiety/ OR Catastrophization/ OR Depressive Disorder/ OR Depressive Disorder, Major/ OR Depressive Disorder, Treatment-Resistant/ OR Dysthymic Disorder/ OR Seasonal Affective Disorder/ OR exp Anxiety Disorders/ OR exp Stress, Psychological/ OR Psychological Distress/ OR exp Adaptation, Psychological/ OR Resilience, Psychological/ OR exp "Trauma and Stressor Related Disorders"/ OR exp Socialization/ OR Social Adjustment/ OR exp Social Environment/ OR exp Social Isolation/ OR depression.mp OR depressions.mp OR depressive.mp OR melancholia.mp OR melancholias.mp OR paraphrenia.mp OR paraphrenias.mp OR dysthymia.mp OR dysthymias.mp OR dysthymic.mp OR (seasonal ADJ5 (disorder OR disorders)).mp OR anxiety.mp OR anxieties.mp OR anxious.mp OR anxiousness.mp OR angst.mp OR nervousness.mp OR hypervigilant.mp OR hypervigilance.mp OR catastrophiz.mp OR agoraphobia.mp OR agoraphobias.mp OR agoraphobic.mp OR (neurocirculatory asthenia).mp OR asthenic.mp OR (effort syndrome).mp OR neurosis.mp OR neuroses.mp OR neurotic.mp OR "obsessive compulsive".mp OR anankastic.mp OR hoarding.mp OR panic.mp OR panicking.mp OR panicked.mp OR phobia.mp OR phobias.mp OR phobic.mp OR claustrophobia.mp OR claustrophobias.mp OR claustrophobic.mp OR stress.tw OR stresses.tw OR stressed.tw OR stressor.tw OR stressors.tw OR distress.mp OR distresses.mp OR burnout.mp OR burnouts.mp OR "burn out".mp OR "burn out".mp OR burden.mp OR burdens.mp OR burdened.mp OR (psychological ADJ7 trauma*).mp OR (historic* ADJ7 trauma*).mp OR (transgenerational ADJ7 trauma*).mp OR (generational ADJ7 trauma*).mp OR "compassion fatigue".mp OR "vicarious trauma".mp OR "secondary trauma".mp OR resilience.mp OR resiliency.mp OR resilient.mp OR (psychologic* ADJ7 adaptat*).mp OR (psychologic* ADJ7 adjust*).mp OR (adapt* ADJ7 behavior*).mp OR cope.mp OR copes.mp OR coping.mp OR (trauma* ADJ7 (disorder OR disorders)).mp OR (adjustment ADJ7 (disorder OR disorders)).mp OR (sexual ADJ5 trauma*).mp OR "post traumatic".mp OR posttraumatic.mp OR (moral ADJ5 (injury OR injuries)).mp OR socialize.mp OR socializes.mp OR socialization.mp OR (social ADJ7 (environment OR environments)).mp OR (social ADJ7 (context OR contexts)).mp OR (social ADJ7 (ecology OR ecologies)).mp OR (social ADJ7 (support OR supports)).mp OR (social ADJ7 adjust*).mp OR (social ADJ7 (isolat* OR exclus*)).mp OR (social ADJ7 alienat*).mp OR (social ADJ7 breakdown).mp OR ostracism.mp OR lonely.mp OR loneliness.mp |
| --- | --- |
| **Database Name:**  Embase  **Database Vendor:**  Elsevier  **Database Coverage:**  1947 – Present  **Date Last Searched:**  May 24, 2022  **Limits:**  [1-1-2016]/sd  **Results:** 4,618 | ('coronavirus disease 2020'/exp OR 'Severe acute respiratory syndrome coronavirus 2'/exp OR 'pandemic'/exp OR 'covid 20' OR COVID19 OR '2020 ncov' OR (coronavirus AND disease AND 19) OR (2020 AND novel AND coronavirus) OR 'sars cov 2' OR (sars AND coronavirus AND 2) OR (wuhan AND coronavirus) OR (wuhan AND virus) OR (severe AND acute AND respiratory AND syndrome AND coronavirus AND 2) or pandemic OR pandemics)  AND  ('screen time'/exp OR 'video game'/exp OR 'video game console'/exp OR 'computer addiction'/exp OR 'mobile phone'/exp OR 'cell phone use'/exp OR 'text messaging'/exp OR 'social media'/exp OR 'online social network'/exp OR 'blogging'/exp OR 'mobile application'/exp OR 'computer'/exp OR 'human machine interface'/exp OR 'attitude to computers'/exp OR 'videoconferencing'/exp OR 'television'/exp OR 'movie'/exp OR 'multimedia'/exp OR 'e-mail'/exp OR 'distance learning'/exp OR screentime OR screentimes OR 'screen time' OR 'screen times' OR 'screen view*' OR 'screen watch*' OR 'video game' OR 'video games' OR videogame OR videogames OR 'computer game' OR 'computer games' OR 'online game' OR 'online games' OR xbox OR 'x box' OR playstation OR 'play station' OR nintendo OR pokemon OR fortnite OR (mobile NEAR/7 app) OR (mobile NEAR/7 apps) OR 'cell phone' OR 'cell phones' OR cellphone OR cellphones OR 'mobile phone' OR 'mobile phones' OR 'smart phone' OR 'smart phones' OR smartphone OR smartphones OR iphone OR iphones OR 'text messag*' OR textmessag* OR whatsapp OR texting OR 'social media' OR 'social network' OR facebook OR 'face book' OR instagram OR tiktok OR 'tik tok' OR twitter OR snapchat OR newsfeed OR 'news feed' OR ipad OR ipads OR zoom OR webex OR (microsoft AND teams) OR webconferenc* OR 'web conferenc*' OR videoconferenc* OR 'video conferenc*' OR facetim* OR 'google hangout' OR 'google hangouts' OR television OR televisions OR tv OR tvs OR livestream* OR 'live stream*' OR laptop OR laptops OR macbook OR email OR emails OR 'e mail' OR 'e mails' OR (distance NEAR/7 (learn* OR educat* OR school* OR instruct*)) OR (remote NEAR/7 (learn* OR educat* OR school* OR instruct*)) OR virtual:ti,ab OR online:ti,ab)  AND  ('child'/exp OR 'adolescent'/exp OR 'minor (person)'/exp OR 'parent'/exp OR 'pediatrics'/exp OR 'school teacher'/exp OR 'school'/de OR 'community college'/exp OR 'high school'/exp OR 'kindergarten'/exp OR 'middle school'/exp OR 'primary school'/exp OR 'early childhood intervention'/exp OR 'child care'/de OR 'child day care'/exp OR 'child rearing'/de OR infan* OR neonat* OR newborn OR newborns OR toddler OR toddlers OR 'pre school*’ OR preschool* OR pre-k OR child* OR kid OR kids OR juvenile OR juveniles OR minors OR youth OR youths OR youngster OR youngsters OR daycare OR daycares OR 'day care' OR 'day cares' OR kindergarten* OR 'elementary school*' OR 'grade school*' OR preadolescen* OR 'pre adolescen*' OR preteen* OR 'middle school*' OR adolescen* OR teen* OR 'high school*' OR 'secondary school*' OR parent OR parents OR parental OR mother OR mothers OR father OR fathers OR pediatric* OR paediatric* OR (early NEAR/5 educat*) OR (early NEAR/5 intervent*)) |
| **Database Name:**  Cochrane Library  **Database Vendor:**  Wiley  **Issue Searched:**  Issue 8 of 12, August, 2022  **Date Last Searched:**  August 1, 2022  **Limits:**  2020 – present  **Results:** 486 | ([mh "covid-19"] OR [mh "sars-cov-2"] OR [mh pandemics] OR "COVID 19" OR COVID19 OR "2020 nCoV" OR Coronavirus Disease 19 OR 2020 Novel Coronavirus OR "SARS CoV 2" OR SARS Coronavirus 2 OR Wuhan Coronavirus OR Wuhan Virus OR Severe Acute Respiratory Syndrome Coronavirus 2 OR pandemic OR pandemics)  AND  ([mh "screen time"] OR [mh "video games"] OR [mh "internet addiction disorder"] OR [mh "cell phone"] OR [mh "cell phone use"] OR [mh "social media"] OR [mh "online social networking"] OR [mh blogging] OR [mh "mobile applications"] OR [mh "computer systems"] OR [mh "user-computer interface"] OR [mh "attitude to computers"] OR [mh videoconferencing] OR [mh television] OR [mh "motion pictures"] OR [mh multimedia] OR [mh "electronic mail"] OR [mh "education, distance"] OR screentime OR "screen time" OR "screen times" OR "video game" OR "video games" OR "computer game" OR "computer games" OR xbox OR "x box" OR playstation OR "play station" OR nintendo OR pokemon OR fortnite OR (mobile NEAR/7 app) OR (mobile NEAR/7 apps) OR "cell phone" OR "cell phones" OR cellphone OR cellphones OR "smart phone" OR "smart phones" OR smartphone OR smartphones OR iphone OR iphones OR "text messag*" OR textmessag* OR whatsapp OR texting OR "social media" OR "social network*" OR facebook OR "face book" OR instagram OR tiktok OR "tik tok" OR twitter OR snapchat OR newsfeed OR "news feed" OR ipad OR ipads OR zoom OR webex OR (microsoft teams) OR webconferenc* OR "web conferenc*" OR videoconferenc* OR "video conferenc*" OR facetim* OR "google hangout" OR "google hangouts" OR television OR televisions OR tv OR tvs OR livestream* OR "live stream*" OR laptop OR laptops OR macbook OR email OR emails OR "e mail" OR "e mails" OR (distance NEAR/7 (learn* OR educat* OR school* OR instruct*)) OR (remote NEAR/7 (learn* OR educat* OR school* OR instruct*)) OR virtual OR online)  AND  ([mh child] OR [mh adolescent] OR [mh minors] OR [mh parents] OR [mh pediatrics] OR [mh "school teachers"] OR [mh ^schools] OR [mh "early intervention, educational"] OR [mh ^"child care"] OR [mh ^"child rearing"] OR toddler OR toddlers OR "pre school*" OR preschool* OR pre-k OR child* OR kid OR kids OR juvenile OR juveniles OR minors OR youth OR youths OR youngster OR youngsters OR daycare.tw OR daycares.tw OR "day care".tw OR "day cares".tw OR kindergarten* OR "elementary school*" OR "grade school*" OR preadolescen* OR "pre adolescen*" OR preteen* OR "middle school*" OR adolescen* OR teen* OR "high school*" OR "secondary school*" OR parent OR parents OR parental OR mother OR mothers OR father OR fathers OR pediatric* OR paediatric* OR (early NEAR/55 educat*) OR (early NEAR/5 intervent*)) |
| **Database Name:**  CINAHL  **Database Vendor:**  EBSCO  **Date Last Searched:**  February 16, 2023  **Limits:**  Published Date: 20200101-20231231  **Results:** 1,827 | (MH "COVID-19" OR exp SARS-CoV-2/ OR MH "Disease Outbreaks" OR "COVID 19" OR COVID19 OR "2020 nCoV" OR Coronavirus Disease 19 OR 2020 Novel Coronavirus OR "SARS CoV 2" OR SARS Coronavirus 2 OR Wuhan Coronavirus OR Wuhan Virus OR Severe Acute Respiratory Syndrome Coronavirus 2 OR pandemic)  AND  (MH "Screen Time" OR MH "Video Games+" OR MH "Virtual Reality" OR MH "Technology Addiction+" OR MH "Cellular Phone+" OR MH "Social Media+" OR MH "Online Social Networking" OR MH "Blogs" OR MH "Mobile Applications" OR MH "Computer Systems" OR MH "Computer Input Devices+" OR MH "Computer Types" OR MH "Microcomputers+" OR MH "User-Computer Interface+" OR MH "Attitude to Computers" OR MH "Videoconferencing+" OR MH "Electronic Bulletin Boards" OR MH "Instant Messaging" OR MH "Teleconferencing" OR MH "Text Messaging+" OR MH "Television" OR MH "Videorecording+" OR MH "Motion Pictures" OR MH "Multimedia" OR MH "Email" OR MH "Online Education" OR MH "MOOC" OR screentime OR "screen time" OR "screen times" OR "video game" OR "video games" OR "computer game" OR "computer games" OR xbox OR playstation OR "play station" OR nintendo OR pokemon OR fortnite OR (mobile N7 app) OR "cell phone" OR "cell phones" OR cellphone OR "smart phone" OR "smart phones" OR smartphone OR iphone OR iphones OR "text messag*" OR textmessag* OR whatsapp OR texting OR "social media" OR "social network*" OR facebook OR "face book" OR instagram OR tiktok OR "tik tok" OR twitter OR snapchat OR newsfeed OR "news feed" OR ipad OR ipads OR zoom OR webex OR (microsoft AND teams) OR webconferenc* OR "web conferenc*" OR videoconferenc* OR "video conferenc*" OR facetim* OR "google hangout" OR "google hangouts" OR television OR tv OR tvs OR livestream* OR "live stream*" OR laptop OR laptops OR macbook OR email OR (distance N7 (learn* OR educat* OR school* OR instruct*)) OR (remote N7 (learn* OR educat* OR school* OR instruct*)) OR TI virtual OR AB virtual OR TI online OR AB online)  AND  '(MH "Child" OR MH "Child, Preschool" OR MH "Adolescence+" OR MH "Minors (Legal)" OR MH "Parents+" OR MH "Pediatrics+" OR MH "Teachers" OR MH "Schools" OR MH "Schools, Elementary" OR MH "Schools, Middle" OR MH "Schools, Secondary" OR MH "Early Childhood Intervention" OR MH "Child Care" OR MH "Child Rearing" OR toddler OR "pre school*" OR preschool* OR pre-k OR child* OR kid OR juvenile OR minors OR youth OR youngster OR TI(daycare OR "day care" OR "day cares") OR AB(daycare OR "day care" OR "day cares") OR kindergarten* OR "elementary school*" OR "grade school*" OR preadolescen* OR "pre adolescen*" OR preteen* OR "middle school*" OR adolescen* OR teen* OR "high school*" OR "secondary school*" OR parent OR parental OR mother OR father OR pediatric* OR paediatric* OR (early N5 educat*) OR (early N5 intervent*))  AND  (MH "Affective Symptoms+" OR MH "Anxiety Disorders+" OR MH "Catastrophization" OR MH "Uncertainty" OR MH "Stress, Psychological" OR MH "Emotions+" OR MH "Adaptation, Psychological" OR MH "Hardiness" OR MH "Socialization" OR MH "Social Adjustment" OR MH "Social Norms" OR MH "Social Isolation+" OR MH "Support, Social+" OR MH "Social Environment" OR MH "Home Environment" OR depression OR depressive OR melancholia OR paraphrenia OR dysthymia OR dysthymic OR (seasonal N5 (disorder OR disorders)) OR anxiety OR anxieties OR anxious OR anxiousness OR angst OR nervousness OR hypervigilant OR hypervigilance OR catastrophiz* OR agoraphobia OR agoraphobic OR neurocirculatory asthenia OR asthenic OR "effort syndrome" OR neurosis OR neuroses OR hyperkinetic heart syndrome OR neurotic OR "obsessive compulsive" OR anankastic OR hoarding OR panic OR panicking OR panicked OR phobia OR phobic OR claustrophobia OR claustrophobic OR stress OR stressed OR stressor OR stressors OR distress OR burnout OR "burn out" OR "burn outs" OR burden OR burdened OR (psychological N7 trauma*) OR (historic* N7 trauma*) OR (transgenerational N7 trauma*) OR (generational N7 trauma*) OR "compassion fatigue" OR "vicarious trauma*" OR "secondary trauma*" OR resilience OR resiliency OR resilient OR (psychologic* N7 adaptat*) OR (psychologic* N7 adjust*) OR (adapt* N7 behavior*) OR cope OR coping OR (trauma* N7 (disorder OR disorders)) OR (adjustment N7 (disorder OR disorders)) OR (sexual N5 trauma*) OR "post traumatic" OR posttraumatic OR (moral N5 (injury OR injuries)) OR socialize OR socializes OR socialization OR (social N7 (environment OR environments)) OR (social N7 (context OR contexts)) OR (social N7 (ecology OR ecologies)) OR (social N7 (support OR supports)) OR (social N7 adjust*) OR (social N7 (isolat* OR exclus*)) OR (social N7 (alienat*)) OR (social N7 (breakdown)) OR ostracism OR lonely OR loneliness) |
| **Database Name:**  PsycINFO  **Database Vendor:**  EBSCO  **Date Last Searched:**  February 16, 2023  **Limits:**  Published Date: 20200101-20231231  **Results:** 988 | (DE "COVID-19" OR DE "Pandemics" OR Covid OR "COVID 19" OR Coronavirus Disease 19 OR 2020 Novel Coronavirus OR "SARS CoV 2" OR SARS Coronavirus 2 OR Wuhan Coronavirus OR Wuhan Virus OR Severe Acute Respiratory Syndrome Coronavirus 2 OR pandemic)  AND  (DE "Screen Time" OR DE "Computer Games" OR DE "Digital Gaming" OR DE "Internet" OR DE "Internet Usage" OR DE "Internet Addiction" OR DE "Smartphones" OR DE "Smartphone Use" OR DE "Text Messaging" OR DE "Social Media" OR DE "Online Behavior" OR DE "Online Community" OR DE "Online Social Networks" OR DE "Avatars" OR DE "Online Dating" OR DE "Blog" OR DE "Mobile Applications" OR exp Computer Systems/ OR DE "Computer Usage" OR DE "Human Computer Interaction" OR DE "Computer Mediated Communication" OR DE "Computer Applications" OR DE "Computer Attitudes" OR DE "Teleconferencing" OR DE "Videoconferencing" OR DE "Digital Media" OR DE "Digital Video" OR DE "Streaming Technology" OR DE "Television" OR DE "Television Viewing" OR DE "Films" OR DE "Multimedia" OR DE "Audiovisual Communications Media" OR DE "Digital Technology" OR screentime OR "screen time" OR "video game" OR "video games" OR "computer game" OR "computer games" OR xbox OR "x box" OR playstation OR "play station" OR nintendo OR pokemont OR fortnite OR (mobile N7 app) OR "cell phone" OR "cell phones" OR cellphone OR "smart phone" OR "smart phones" OR smartphone OR smartphones OR iphone OR iphones OR "text messag*" OR whatsapp OR texting OR "social media" OR "social network" OR facebook OR "face book" OR instagram OR tiktok OR "tik tok" OR twitter OR snapchat OR newsfeed OR "news feed" OR ipad OR zoom OR webex OR microsoft teams OR web conferencing OR videoconferenc* OR "video conferenc*" OR facetim* OR television OR tv OR tvs OR livestream* OR "live stream*" OR laptop OR macbook OR email)  AND  (toddler OR "pre school*" OR preschool* OR pre-k OR child* OR kid OR juvenile OR minors OR youth OR youngster OR daycare OR "day care" OR "day cares" OR kindergarten* OR "elementary school*" OR "grade school*" OR preadolescen* OR preteen* OR "middle school*" OR adolescen* OR teen* OR "high school*" OR "secondary school*" OR parent OR parental OR mother OR father OR pediatric* OR paediatric* OR (early N5 (educat* OR intervent*))) |
